# Illusory Distance Modulates Perceived Size of Afterimage despite the Disappearance of Depth Cues

**DOI:** 10.1371/journal.pone.0159228

**Published:** 2016-07-08

**Authors:** Jiehui Qian, Shengxi Liu, Quan Lei

**Affiliations:** 1 Department of Psychology, Sun Yat-Sen University, Guangzhou, Guangdong, China; 2 Department of Psychology, University of Minnesota Twin-Cities, Minneapolis, MN, United States of America; University of Akron, UNITED STATES

## Abstract

It is known that the perceived size of an afterimage is modulated by the perceived distance between the observer and the depth plane on which the afterimage is projected (Emmert’s law). Illusions like Ponzo demonstrate that illusory distance induced by depth cues can also affect the perceived size of an object. In this study, we report that the illusory distance not only modulates the perceived size of object’s afterimage during the presence of the depth cues, but the modulation persists after the disappearance of the depth cues. We used an adapted version of the classic Ponzo illusion. Illusory depth perception was induced by linear perspective cues with two tilted lines converging at the upper boundary of the display. Two horizontal bars were placed between the two lines, resulting in a percept of the upper bar to be farther away than the lower bar. Observers were instructed to make judgment about the relative size of the afterimage of the lower and the upper bars after adaptation. When the perspective cues and the bars were static, the illusory effect of the Ponzo afterimage is consistent with that of the traditional size-distance illusion. When the perspective cues were flickering and the bars were static, only the afterimage of the latter was perceived, yet still a considerable amount of the illusory effect was perceived. The results could not be explained by memory of a prejudgment of the bar length during the adaptation phase. The findings suggest that cooccurrences of depth cues and object may link a depth marker for the object, so that the perceived size of the object or its afterimage is modulated by feedback of depth information from higher-level visual cortex even when there is no depth cues directly available on the retinal level.

## Introduction

Size constancy is a renowned phenomenon that demonstrates perceptual size stabilization accounting for the effect of viewing distance on retinal image size. For example, Emmert’s law demonstrates that an afterimage appeared to increase in size when projected to a greater distance, and vice versa [1, 2]. It has long been proposed that perceived size of an object is a function of its retinal image size and the perceived distance between observer and the object, a so-called size-distance invariance hypothesis (SDIH) [2–4]. If the perceived distance goes wrong, size illusions occur. Previous research suggests that a number of size illusions, such as the Ponzo and moon illusions, can be attributed to an underlying mechanism of compensating for retinal image changes associated with misperceived distance [5–11]. For example, one of the most influential explanations for the Ponzo illusion is the ‘misapplied size constancy’ theory, proposed by Richard Gregory [7, 8]. It suggests that in order to maintain size constancy in natural three-dimensional scenes, near objects are perceptually reduced in size and distant objets are perceptually enlarged. However, when there is no physical change in distance but an illusory one is induced by depth cues, such as the converging lines which serve as a linear perspective cue in the Ponzo figure, mis-application of the size constancy mechanism would lead to distortions in perceived size of objects.

The compensation mechanism seems to not only work for real objects, but also for afterimages. Dwyer et al. [12] showed that perceived size of afterimage increased as the apparent distance increased, consistent with Emmert’s law. In their study, size judgments of afterimages projected into the Ames room were compared with control conditions in which both the actual and the apparent distances of afterimage projection in Ames room were reproduced. The authors suggested that the processes involved in making size judgments of afterimages were similar to that of ‘real world’ objects. This also holds true for proprioceptive or haptic depth cues [13], as well as in virtual environment [14]. Furthermore, study showed that perceived shape of afterimage changed as it was projected on an apparently slanted surface [15]. These studies indicate that real objects and afterimages may function similarly in size perception.

A recent fMRI study on afterimage shows that the retinal signals reaching V1 are modulated by distance information in a fashion that reflects the implementation of size-constancy mechanism [16]. By employing a similar paradigms as in Emmert’s law, the authors found that the larger the perceived size of the afterimage, the larger the retinotopic activation in V1, while the size of the retinal image remains the same. Feedback from higher visual areas seems to provide distance information for scaling the cortical activity, supported by neuroimaging studies and modeling work [17–19]. In addition, single cell recordings in awake and anesthetized monkeys found distance- dependent size tuned cells along the ventral pathway from visual cortical area V1, V2 and V4 [20] leading to inferotemporal (IT) cortex [21]. These evidences indicate that perceived size of both object and afterimage is mediated by V1 activation, which may receive feedback of distance information from higher-level visual cortex.

While research employing Emmert’s paradigm suggests that afterimage size perception is subject to distance-scaling as is an object, it is less clear whether afterimage could serve as an effective depth cue modulating the size perception on object. Studies show that depth may be seen in afterimages in line stereograms and random-dot stereograms [22–24]. Cormack found almost perfect depth constancy up to 27 m by using a pair of disparate afterimages as the test object [25]. The use of afterimage overcame the difficulty of keeping disparity constant over the changes in vergence or accommodation, but the study was also criticized for only reporting the results derived from the use of the depth probe, for which might provide a standard when comparing different sources of depth information. Despite of the dispute, researchers generally accept that good stereo depth can be obtained by afterimages. However, could depth perception be induced by afterimages of monocular depth cues? If yes, do the depth cues differ in strength between objects and their afterimages? Solving these questions may contribute to the understanding of top-down modulation of distance information on size perception.

The Ponzo illusion is one of the most popular and well-known illusions allowing depth perception from monocular pictorial depth cues. However, few research has examined the afterimage effect of the Ponzo illusion. Could the Ponzo illusion be replicated by using afterimages as either the inducing depth cues, or the target possibly affected by the inducers, or both? How does the illusion produced in these conditions differ from the original one? Specifically, how is the strength of the illusion affected by different depth cues? In order to investigate these questions, we adopted an experimental design similar to Sperandio et al. [26], in which an Ebbinghaus—Titchener figure with flickering surrounding inducing annuli and a static inner test annulus was used to examine the perceived size of afterimage. Their results suggested that high-level cognitive processes play an important role on the perceived size of an afterimage beyond the retinal level. In our study, by adoption of an afterimage version of the Ponzo illusion, we investigated whether size perception of afterimage of the illusion could be explained by the misapplied size constancy, which might further shed light on the underlying mechanism of distance-scaling on size perception. We adapted Sperandio et al.’s [26] research paradigm to investigate the Ponzo illusion. Observers were first adapted to linear perspective cues, then tested the effect on the target stimuli. Depth cues, as well as targets, were presented as either static or flickering. The resulting differences observed among these experimental conditions suggest that object might be linked with its associated depth cues by a depth marker, so that the perceived size of both the object and its afterimage could be modulated by feedback of depth information immediately after the disappearance of depth cues.

## Methods

### Observers

Five observers (3 males) with normal or corrected visual acuity took part in all five experiments for pay. Four of the observers were naive to the purpose of the study; two were experienced psychophysical observers. Observers were trained for a short time (2–5 min) to make sure that he or she was able to see an afterimage after 30-seconds adaptation to the stimuli. This research was approved by the Sun Yat-Sen University Institutional Review Board (IRB). Written informed consent was provided by each participant prior to the experiment.

### Apparatus

The stimuli were displayed on a uniform gray background (51.5*cd*/*m*^2^) and viewed on a 23-inch HP proDisplay P231 monitor. The display resolution was set to 1600 x 900 pixels, with a refresh rate of 60 Hz. The typical viewing distance was 57 cm. Observers seated in a dark room to complete the whole experiments.

### Stimuli

**Fig 1:**
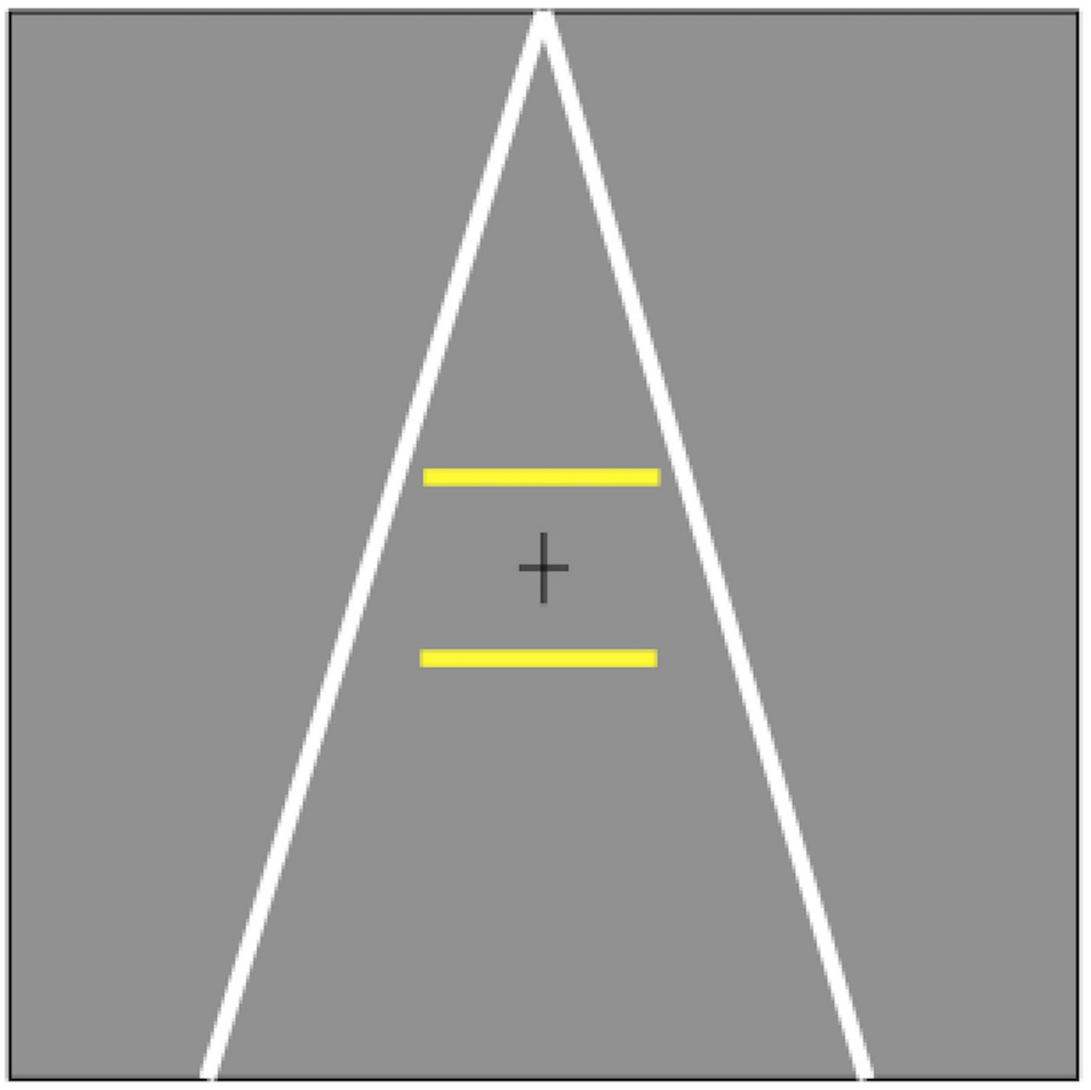
Stimuli. Two converging lines serves as linear perspective cues. The Ponzo illusion is that the lower bar is perceived to be shorter than the upper bar even when their length is the same (as shown in the figure).

An adapted version of the Ponzo illusion was employed in the experiments. Depth perception was induced by two tilted lines that converged at the upper boundary of the display. The length of the lines was 28°, with a width of 0.2°. Two bars were placed at the center of the screen, one above the fixation cross, one below (Fig 1). The length of the upper bar was fixed at 6°, while the lower bar varied in length in order to measure the illusory effect.

### Experimental Procedure

The experimental design in our study was similar to Sperandio et al. [26]. For all five experiments, each trial consisted of two phases, first an adaptation phase, then an afterimage phase. The adaptation phase lasted for 30 seconds. In this phase, the two converging lines, which serve as the perspective depth cue, were either flickering (flicker mode) or remained static (static mode) depending on the different experimental purposes, except for Experiment 1a, where no depth cue was introduced in the adaptation phase. In the flicker mode, the lines flickered between white (103*cd*/*m*^2^) and black (.14*cd*/*m*^2^) at a rate of 4Hz, with an average luminance equal to the background. Because the cortical processes underlying the afterimage generation are too slow to follow the swift changes in local luminance, no polarity-specific adaptation occurs after a prolonged exposure to such flickering stimuli [27]. In the static mode, the lines remained white during the 30 secs presentation. Observers were instructed to maintain their fixation on a small black cross presented in the center of the screen during the whole adaptation phase. The distance of the central two bars to the fixation were the same, 4°. Both bars were yellow (98.2*cd*/*m*^2^) during the 30 secs (except for Experiment 4, where no bars were presented, and Experiment 5, where the bars flickered between white and black at 4Hz). The entire display disappeared after 30 secs presentation.

In the following afterimage phase, a uniform gray background with a fixation cross in the center was presented. After seeing the afterimage (or physical stimuli in Experiment 2a, 4 and 5), observers were instructed to press the left key when they perceive the lower bar to be shorter than the upper bar and press the right key for the opposite, while keeping their fixation in the center cross. If no difference was perceived between the upper and the lower bar, they were instructed to equally distribute ‘no difference’ answer between the right and the left keys. We encouraged them to discriminate even a tiny slight difference between the two bars, and to try their best to avoid ‘no difference’ answer. They were also asked to ignore any possible prejudgment of the lengths of the bars during the adaptation phase, only to compare the relative lengths of the bars’ afterimage. After the key press, a blank screen was shown for 4 secs in order for the afterimage to fade away, and observers were allowed free viewing during this period. The lower bar varied from 5.7° to 7.2°, with an increment of 0.214°, resulting in 7 levels of physical length. Each length was tested for five trials, resulting in a total of 35 trials for each experiment. The order of all trials was randomized. Observers received a randomized order of all five experimental conditions.

### Data Analysis

The effect of the illusion was defined as the percentage of the perceived decrease of the lower bar length. To compensate for the illusory effect, the physical length of the lower bar had to increase by the same amount in order for observers to perceive the upper and the low bars as equal. For data analysis, the probability of the lower bar being perceived as longer was plotted against the percent of physical increase of the lower bar length. The data was fitted with a Weibull function. The illusory effect was calculated as the point of subjective equality (PSE), which was derived as the percent of physical increase of the lower bar length that corresponds to a 50% probability on the Weibull curve. A PSE greater than zero indicates an underestimation of the lower bar length and a PSE less than zero indicates the opposite. Data available in S1 Data.

## Results

### Experiment 1: Effect of depth cues on perceived afterimage size of bars

**Fig 2:**
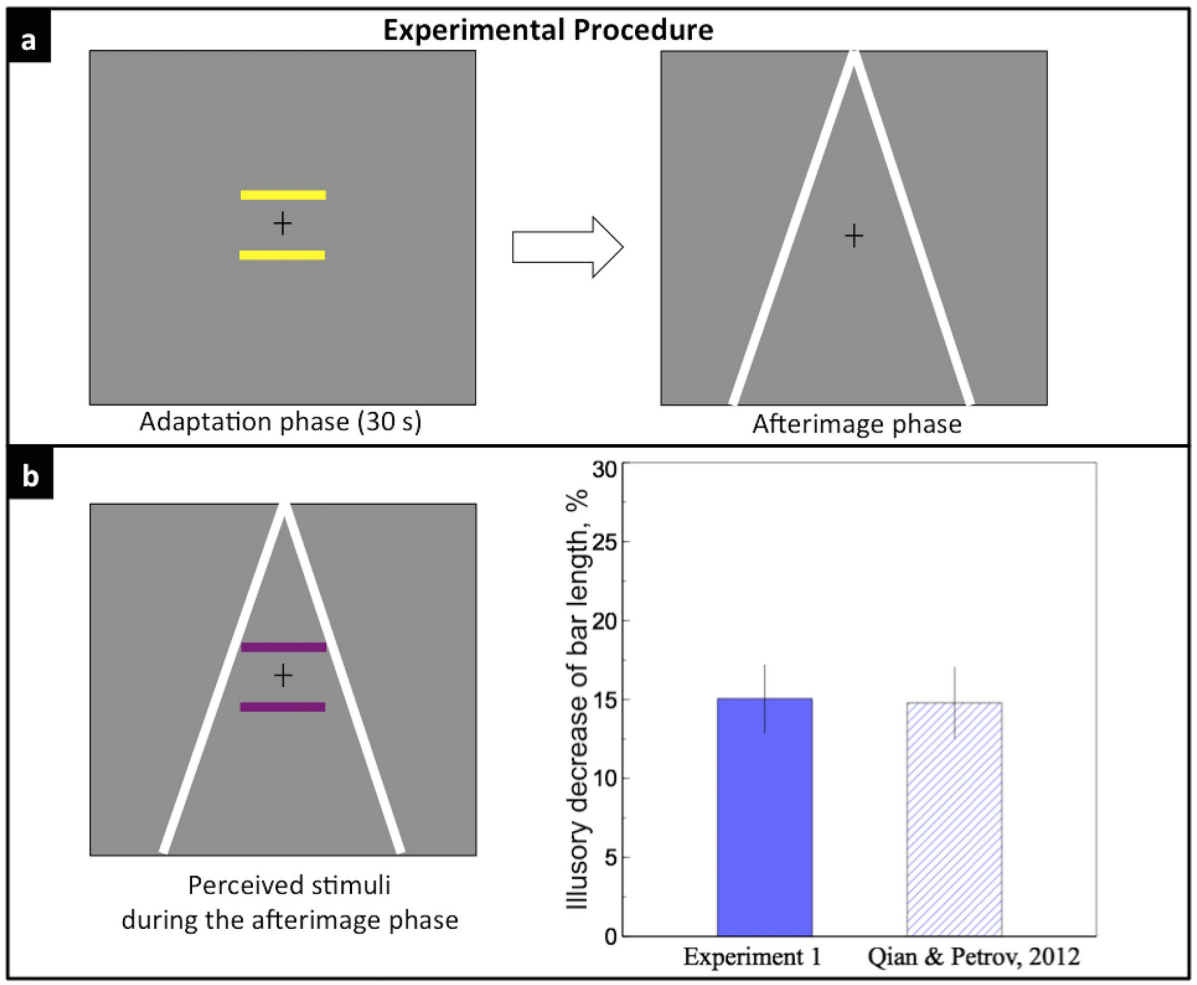
Experiment 1. a) Two bars were presented during the 30-s adaptation phase; two converging lines were shown during the afterimage phase. b) Left: the perceive stimuli during the afterimage phase; Right: the purple bar indicates the mean illusory decrease of the lower bar length in Experiment 1, 15.05%; the shadowed bar indicates the illusory effect of a similar size-distance illusion reported in our previous study [10]. Error bar represents one standard error here and in other figures.

The purpose of this experiment was to test the effect of the explicit depth cues, such as linear perspective cues, on perceived afterimage size. In the adaptation phase, only two test bars were presented for 30 secs; in the afterimage phase, two converging lines were shown on the display (Fig 2a). If the Ponzo illusion paradigm worked on afterimages of the bars, observers would perceive the lower bar as being shorter.

Observers reported being able to see purple afterimages of the bars 3–5 s after the adaptation phase ended. The afterimage of the bars persisted for about 8 s. Fig 2b shows that the mean illusory effect was 15.05 ± 2.16%. In other words, the physical length of the lower bar had to be increased by about 15% in order for the observers to perceive the two bars as equal. Our previous study showed a similar size-distance illusion, where the perceived size of the disks increased by about 15% as the illusory distance of the disks appeared to increase, even when the angular size of the disks remained the same [10]. The result is also consistent with previous research on the Ponzo illusion, showing a typical 10% to 15% illusory effect with two converging lines settings [28, 29]. This experiment shows that depth cues can affect the perceived size of an object as well as its afterimage as previous research has shown.

### Experiment 2a: Effect of depth cues’ afterimage on perceived size of bars

**Fig 3:**
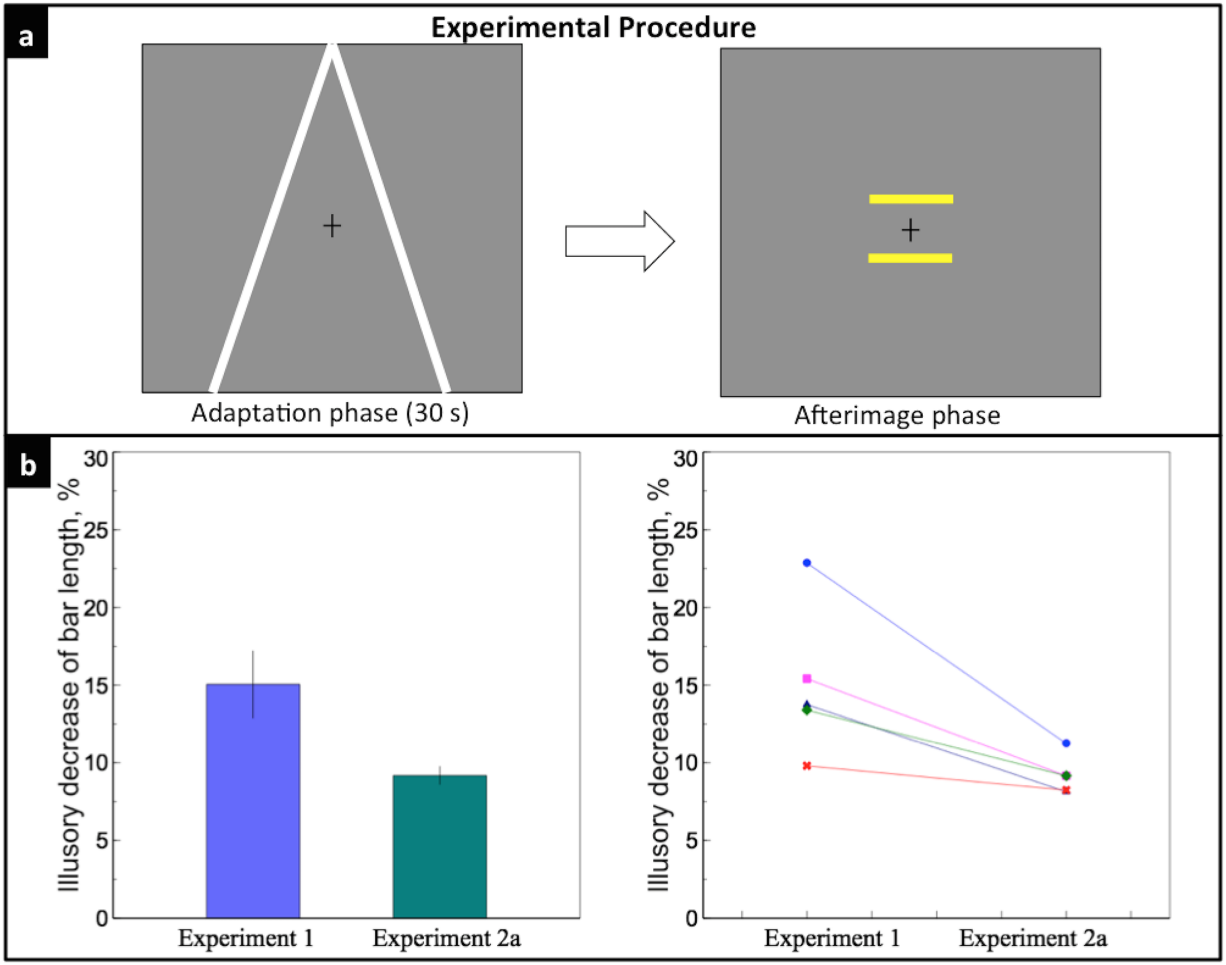
Experiment 2a. a) Two lines were presented during the 30-s adaptation phase; two bars were shown during the afterimage phase. b) Left: the dark green bar indicates the mean illusory decrease of the lower bar length in Experiment 2a, 9.19%. Results of Experiment 1 were replotted here for comparison. Right: individual data of the five observers. Each observer was indicated by different colors and symbols. There was a significant difference between the illusory decrease of bar length of Experiment 1 and that of Experiment 2a (*p* = 0.02).

In Experiment 1, we showed that depth cues could affect the perceived afterimage size of an object. Alternatively, we wanted to know whether an afterimage of the depth cues could produce the same of illusory effect. Therefore in Experiment 2a, the converging lines were presented for 30 secs in the adaptation phase; in the afterimage phase, only two test bars were presented (Fig 3a).

Fig 3b shows the illusory decrease of the lower bar length. On average, the illusory effect was 9.19 ± 1.26%. There was a significant difference between the illusory effect of Experiment 1 and that of Experiment 2a (*t*(4) = 3.56, *p* = 0.02). Individual data shows that the illusory effect was stronger in Experiment 1 for all five subjects. The results are predictable, since the afterimage of the two converging lines provided weaker depth cues in Experiment 2a, compared with Experiment 1 where explicit depth cues were present in the afterimage phase.

### Experiment 2b: Effect of depth cues afterimage on perceived afterimage size of bars

**Fig 4:**
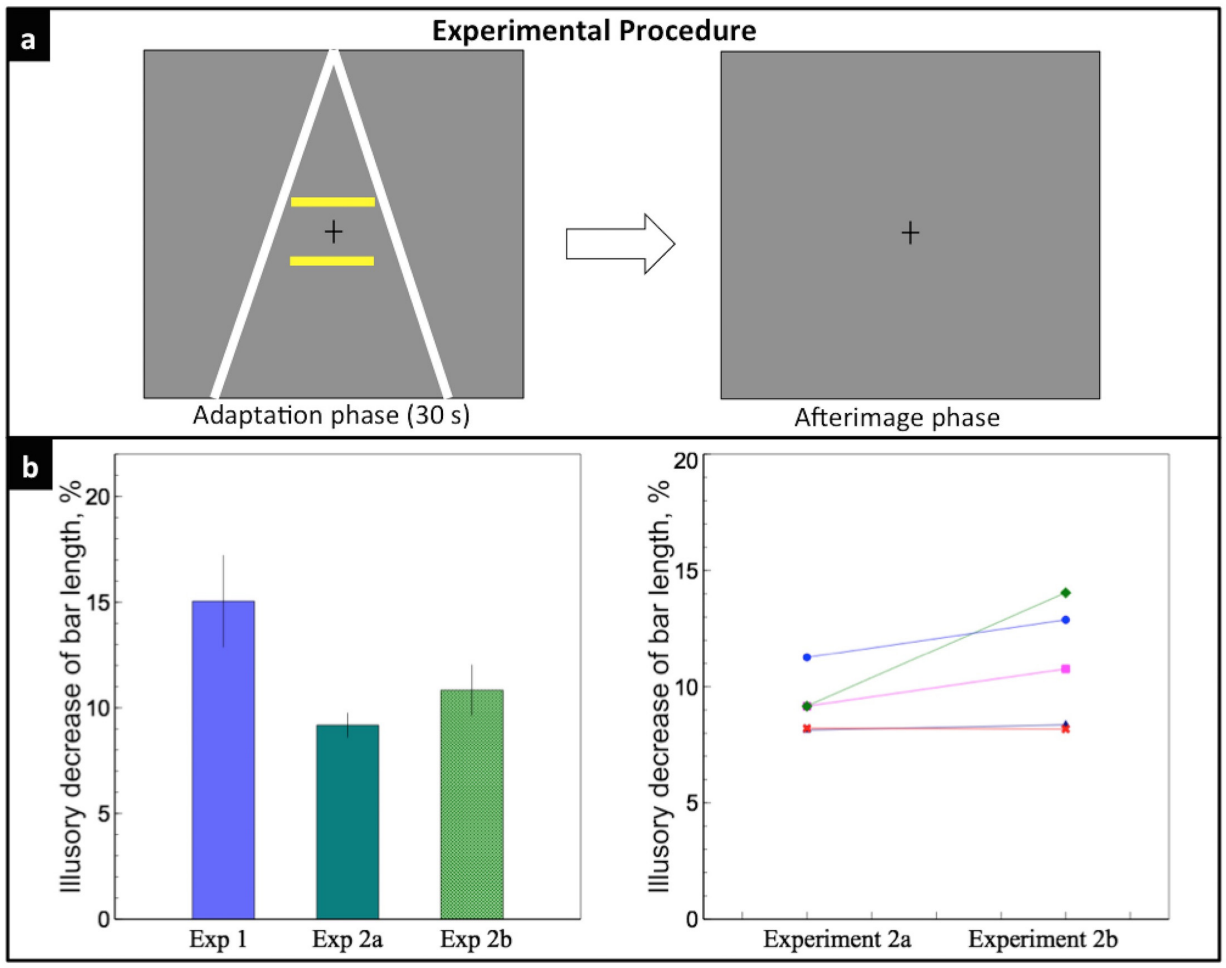
Experiment 2b. a) Two lines and two bars were presented during the 30-s adaptation phase; a gray background was shown during the afterimage phase. b) Left: the light green bar indicates the mean illusory decrease of the lower bar length in Experiment 2b, 10.84%. Right: individual data of the five observers (colors and symbols were consistent with Fig 3b). There was no significant difference between the illusory decrease of bar length of Experiment 2a and that of Experiment 2b (*p* = 0.13).

In Experiment 2b, we tested whether the afterimages of the depth cues could affect the perceived afterimage size of the bars. In the adaptation phase, both the lines and the bars were presented for 30 secs; in the afterimage phase, the display was replaced with a blank screen (Fig 4a).

Fig 4b shows the average illusory decrease of the lower bar length was 10.84 ± 1.18%. There is no significant difference between the illusory effect of Experiment 2a and that of Experiment 2b (*t*(4) = 1.90, *p* = 0.13), suggesting that the same amount of illusion could be perceived on either the physical bars or the afterimages of the bars, as long as the same depth percept resulted from the same depth cues are observed.

### Experiment 3: Effect of flickering depth cues on perceived size of afterimage

**Fig 5:**
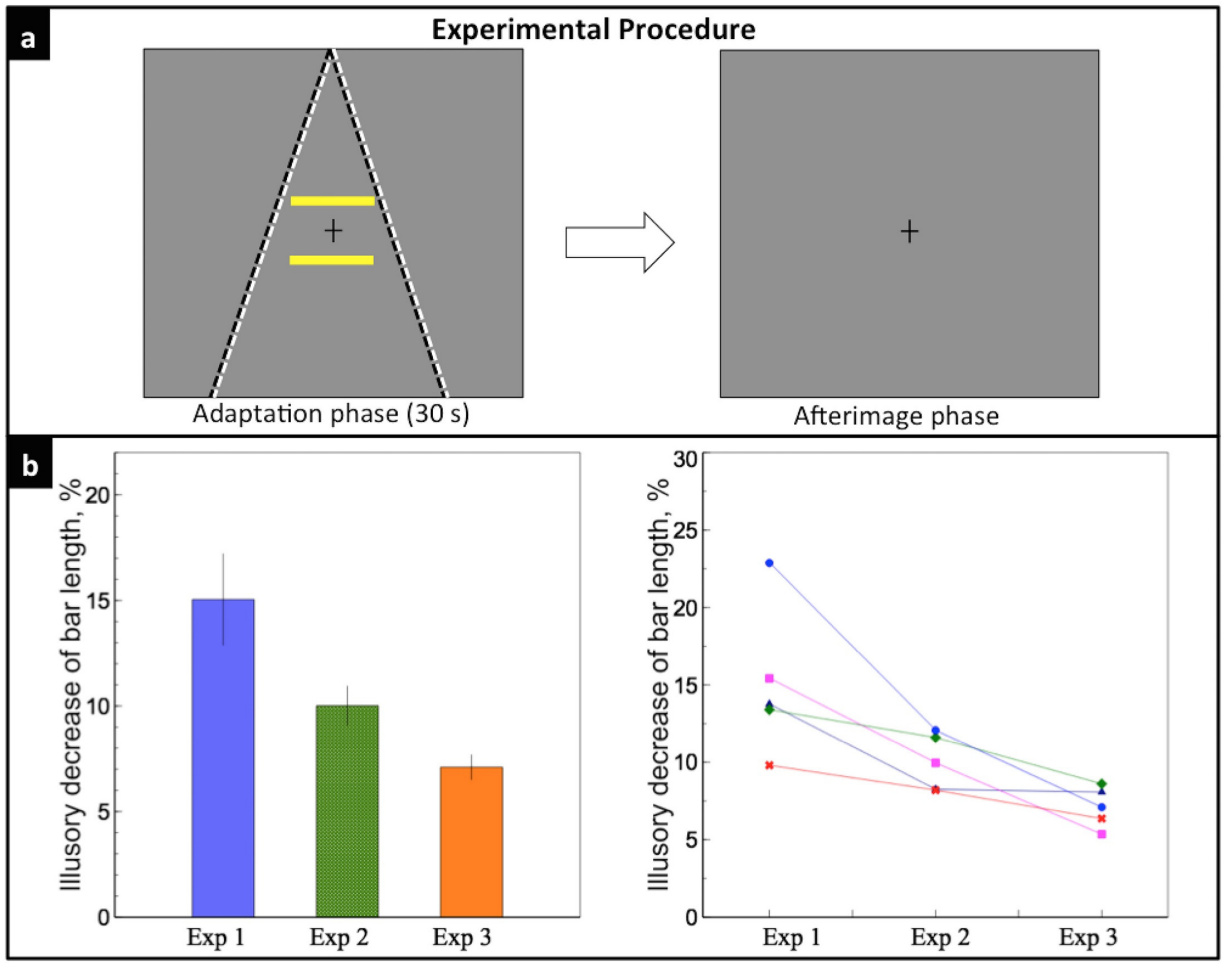
Experiment 3. a) Two lines flickered at 4 Hz and two bars remained presented during the 30-s adaptation phase; a gray background was shown during the afterimage phase. b) Left: the orange bar indicates the mean illusory decrease of the lower bar length in Experiment 3, 7.10%; the green bar indicates an average illusory effect of Experiment 2a and 2b, 10.02%. Right: individual data of the five observers (colors and symbols were consistent with Fig 3b). There was a significant difference between the illusory decrease of bar length of Experiment 2a, 2b and Experiment 3 (*p* = 0.03).

In this experiment, we tested whether the flickering depth cues could induce a similar illusory effect as shown in Experiment 2b. This experiment was identical to Experiment 2b, except that in the adaptation phase, the two lines were flickered at 4 Hz and the bars remained static for 30 secs (Fig 5a). Since counterphase-flickering stimuli do not generate a detectable afterimage [26, 27], no depth cues were available during the afterimage phase.

Observers reported that only the purple afterimages of the bars were seen in the afterimage phase, no afterimage of the depth cues was perceived. The mean illusory decrease of the lower bar length was 7.10 ± 0.58%. Comparison of the illusory strength among Experiment 1, 2 and 3 is shown in Fig 5b. There was a significant difference between the illusory effects of Experiment 2a, 2b and that of Experiment 3 (repeated measure ANOVA, *F*(2, 8) = 7.72, *p* = 0.01). Individual data shows that the illusory effect decreased for all five subjects. However, the results are surprising since there was no depth cue available during the afterimage phase yet the observers still perceived the illusion.

### Experiment 4: Effect of flickering depth cues on perceived size of bars

**Fig 6:**
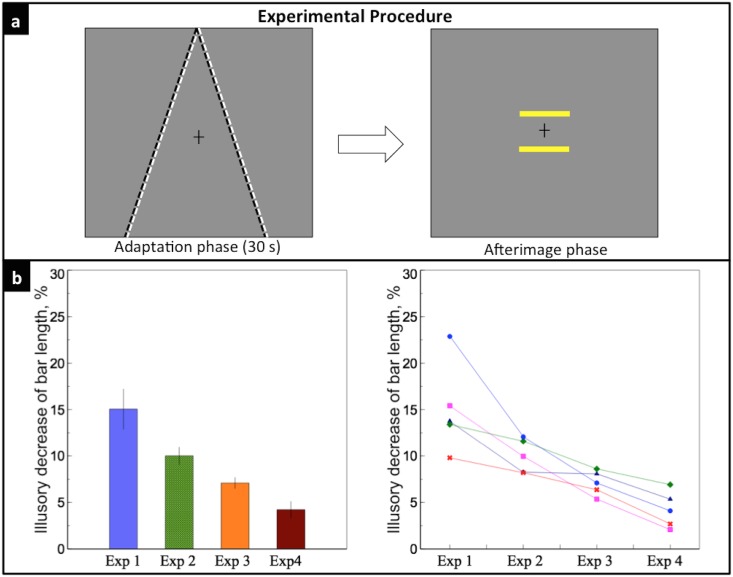
Experiment 4. a) Two lines flickered at 4 Hz during the 30-s adaptation phase; two bars were shown during the afterimage phase. b) Left: the dark red bar indicates the mean illusory decrease of the lower bar length in Experiment 4, 4.23%. Right: individual data of the five observers (colors and symbols were consistent with Fig 3b). There was a significant difference between the illusory decrease of bar size of Experiment 4 and Experiment 3 (*p* = 0.001).

One possible explanation for Experiment 3 is that the flickering depth cues induced a subthreshold aftereffect even though observers could not detect a distinguishable afterimage. To test this possibility, we conducted Experiment 4. Instead of showing two bars during the adaptation phase, bars were shown after observers adapted with flickering depth cues (Fig 6a). If the flickering stimuli induced an aftereffect, we would observe the same illusory effect on the two bars as on the afterimage.

Fig 6b shows that the mean illusory decrease of the lower bar length was 4.23 ± 0.88%. This small illusory effect suggests that perceived size of the bar might be affected by a possible aftereffect of the flickering perspective cues. Individual data shows that the illusory effect diminished for all five subjects. There was a significant difference between the illusory effect of Experiment 4 and that of Experiment 3 (*t*(4) = 8.56, *p* = 0.001). This indicates that the mere residual aftereffect cannot fully explain the significantly greater illusory effect observed in Experiment 3. There may be other factors contributing to the illusion.

### Experiment 5: Effect of the memory of depth cues on perceived size of bars

**Fig 7:**
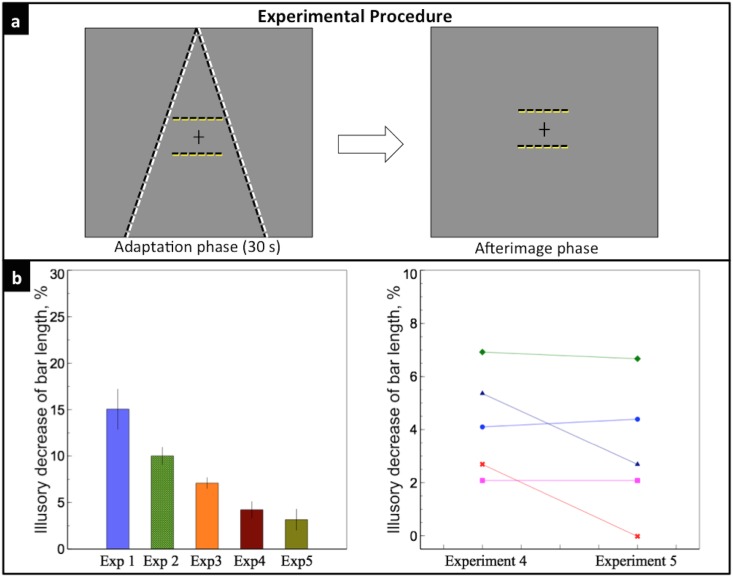
Experiment 5. a) Two lines and two bars flickered at 4 Hz during the 30-s adaptation phase; only flickering bars were shown during the afterimage phase. b) Left: the olive bar indicates the mean illusory decrease of the lower bar length in Experiment 5, 3.16%. Right: individual data of the five observers, indicated by different colors and symbols. There was no significant difference between the illusory decrease of bar length of Experiment 5 and 4 (*p* = 0.19).

Since both the bars and the lines were shown during the adaptation phase in Experiment 3, another possible explanation is that the memory of a prejudgment on the length of the bars during the adaptation phase affected observers’ judgment during the afterimage phase. To rule out this possibility, we introduced a full flicker version of the Ponzo illusion in Experiment 5. In the adaptation phase, the lines flickered between white and black and the bars flickered between yellow and black, both at a rate of 4Hz; in the afterimage phase, only the flickering bars were shown (Fig 7a). If memory contributed to the illusory effect, we expect to observe the same pattern of the results as in Experiment 3.

Fig 7b shows that the mean illusory decrease of the lower bar length was 3.16 ± 1.13%. There was no significant difference between the illusory effect of Experiment 5 and that of Experiment 4 (*t*(4) = 1.60, *p* = 0.19). The results indicate that a memory-based prejudgment cannot explain the effect observed in Experiment 3. The small illusory effect may be caused by aftereffect of the flickering perspective cues on perceived size of the bars.

## Discussion

Current study investigated the effect of adaptation to linear perspective cues on perceived size of afterimage. Experiment 1 and 2 showed that the Ponzo illusion could be observed with afterimage of either the test bars or the depth cues, or both. In Experiment 3, we found that flickering depth cues induced an illusory effect, even though no detectable afterimage of depth cues was observed. Experiment 4 and 5 tested the possible explanations for the results of Experiment 3.

The mean illusory effect was about 15% if the depth cues were explicitly shown (Experiment 1), and was about 10% if the afterimages of the depth cues were observed (Experiment 2a and 2b). No significant difference was found between the conditions that tested the actual bars and that tested the afterimages of the bars (*p* = 0.13), suggesting that the illusory effect does not differ between the real object or its afterimage, as long as the test target is viewed with the same depth percept resulted from the same depth cues. In Experiment 3, no inducer or afterimage of the inducers was available during the afterimage phase, yet the illusion was still observed. This result was in accordance with Sperandio et al.’s finding on afterimage of Ebbinghaus—Titchener illusion [26]. In their study, observers first adapted to flickering surrounding inducing annuli and a static inner test annulus, then estimated the size of the test annulus after adaptation. The illusion occurred even when the flickering surrounding annuli had not generated afterimage. These findings indicated that perception of afterimages was not a simple consequence of bleaching photoreceptor pigments in the retina but also depended on high-level cognitive processes. Furthermore, the authors suggested that lateral inhibition operating at the retina level did not play a major role in the illusory effects of Ebbinghaus—Titchener figures, and that post-retinal factors might be responsible for the modulation in perceived size. This was in accordance with an fMRI study on the Ebbinghaus—Titchener illusion, which showed that the retinotopic coding in V1 was modulated by perceived size [30].

Experiment 5 showed that there was no carry-over memory effect. One might argue that judgment made on the afterimages of the test bars in Experiment 3 is different from judgment made on the real test bars in this experiment. Presumably, a memory of the prejudgment on the bars’ lengths may have a greater influence on the perceptually weaker afterimage than on the real object. However, if this is the case, we should observe a greater illusory effect in Experiment 2b than in Experiment 2a, since likewise judgments were made on the afterimage of the test bars in Experiment 2b and on the real test bars in Experiment 2a. Non-significant difference between the results of Experiment 2a and 2b suggests that object and its afterimage may be treated equally in terms of visual processing, even though afterimage might be perceived less distinctively. This is consistent with several studies on size and shape judgments on afterimages, which suggest that perception of an afterimage is similar to perception of real world objects [12, 15, 26].

The results of Experiment 4 suggest a subthreshold aftereffect of the flickering depth cues might influence the illusory effect. Another possible explanation is that the image of the test bars was involuntarily integrated with the memory of the previously viewed inducing depth cues, which resulted in the illusion. Study showed that when the Ponzo illusion figure was divided into its individual components and sequentially encoded into visual working memory (VWM), the temporally separated components were involuntarily integrated and therefore triggered the illusion [31]. The study found an illusory effect of about 5%—6%. Although we did not ask observers to perform any task that required to hold the inducing depth cues in the VWM, it is possible that the inducers remained in the VWM after prolonged viewing. The illusory effect was slightly weaker (about 4%) in our experiment, this might be due to that no explicit working memory task was performed. Nevertheless, neither a subthreshold aftereffect nor involuntary integration in VWM could fully explain the results found in Experiment 3, since a significant difference was found between the results of Experiment 3 and that of Experiment 4 (*p* = 0.001). This indicates that other factors besides these possible explanations must contribute to the illusion.

Estimation of viewing distance is crucial in order for observers to perceive object’s veridical size. Past research shows that depth cues do not work in an all-or-none fashion but vary in strength given different situations. Oculomotor depth cues work in a short range (typically less than 2 m), while pictorial depth cues might dominate the depth percept at a larger distance [32–34]. Leibowitz, Brislin, Perlmutrer, and Hennessy (1969) found that different depth cues could affect the magnitude of size constancy. They measured the overestimation of object size at varying distances by measuring the magnitude of the Ponzo illusion, and the results showed that the illusory effect was about 45% for three-dimensional actual scenes compared to 30% for two-dimensional photographs of the same scene. It is consistent with the Bayesian cue integration view that in perceptual models cues are weighted and combined according to their reliability [35]. In our study, afterimage of linear perspective cues may signal the distance information. However, in Experiment 3, that the illusion occurred without explicit depth cues may suggest other depth indicators existing. We propose that the co-occurrences of depth cues and object may provide a depth marker for the object, so after a prolonged exposure to the object, the perceived size of the object’s afterimage is modulated by feedback of distance information even when there is no retinal-level depth cues available. It is ecologically plausible that human involuntarily encode and store the location information of an object, including its perceived distance, for convenience. In Experiment 5, even though the inducers and the bars co-occurred during the adaptation phase, it is not definitive whether the test bars shown in the afterimage phase were continuation of the original bars or a new pair of flickering bars. Hence, the depth marker for the original bars may not function. However, in Experiment 3, the afterimages of the bars was obviously continuation of the original ones, therefore the associated distance information may still apply. This causes a significantly stronger illusory effect on the afterimage. For this reason, we suggest that an object might be linked with its associated depth cues by a depth marker; after the disappearance of depth cues, this depth marker persists and continues to provide distance information to modulate the perception of the afterimage, which is treated as a continuation of the earlier object by the visual system.

Many researchers found that afterimages may have visual functions just as ‘real world’ objects [12, 15]. For example, Kirschfeld reported a common neural correlate of visual consciousness for images and afterimages [36]. Previous research has shown that afterimages have a retinal locus [37–39], and the generation of color afterimages is mediated at least in part by the primary visual cortex (V1) [40, 41]. In addition, several fMRI studies found that when observing objects against a receding hallway background, activation in V1 are modulated by distance percept, suggesting size-distance scaling mechanisms [17, 18]. Sperandio et al. (2012) examined brain activation using fMRI when participants viewed afterimages on surfaces placed at different distances. They found that the retinotopic activation in V1 was associated with the perceived size of the afterimage, indicating feedback of depth information from other brain areas. This feedback may come from higher visual cortex along the dorsal pathway, such as the lateral intraparietal cortex (LIP), where three-dimensional (3D) spatial representation is supposed to be constructed [42]. In our study, perceived size of an object and its afterimage can be affected by apparent distance. Furthermore, the afterimage of linear perspective cues not only seems to convey distance information to the visual system, but also to function as if it was real in space. The results suggest that the generation of afterimage is more than a simple adaptation of the photoreceptors on the retina, it also depends on higher cognitive processes similar to object perception. Research also shows that higher-level visual functions, such as attention, filling-in, awareness and contextual modulation, have effects on afterimage perception [26, 43–46]. Therefore, it is not surprising that the perception of afterimage is subject to modulation by higher-level feedback of distance information, which may be signaled by concurrent depth cues or by depth marker previously associated with the object.

Finally, a number of possible explanations have been suggested for the Ponzo illusion. Besides the most influential explanation that attributed the illusory effect to mis-compensation of size constancy mechanism—the ‘misapplied size constancy’ theory, other possibilities have also been brought into attention. For example, the ‘tilt constancy’ theory states that the illusion is caused by the misperception of orientation induced by local visual cues [28]; the ‘integrative field’ theory suggests that when comparing the two bars in the Ponzo figure, the visual system creates an attentive field that provides various perceptual weights to different parts of the figure, and the illusory effect results from integrating the focal line lengths by weights [47]. In most experimental conditions, all three theories could equally explain the results. For example, in Experiment 4, either a subthreshold aftereffect or involuntary integration in VWM allows both the inducer and the test bars temporarily available in a visuospatial sketchpad, hence misperception of distance, misperception of orientation, or integration of focal visual information could result in the illusion. However, the latter two theories cannot explain the results of Experiment 3. Therefore, our study suggests that the tilt constancy or the integrative field theory alone could not sufficiently explain the underlying mechanism of the Ponzo illusion.

## Conclusion

We employed an adapted version of the Ponzo illusion to study the effect of pictorial depth cues on afterimage. We found that the illusory distance not only modulates the perceived size of afterimage during the presence of the depth cues, but also persists its modulation after the disappearance of the depth cues. The findings suggest that the perceived size of the object and its afterimage may be modulated by higher level cognitive processing in the brain, possibly via feedback of depth information from higher-level visual cortex. Further neurophysiological research may shed lights on the neural mechanisms in afterimage perception.

## Supporting Information

S1 Data Data for Experiment 1– 5. (SAV)
